# Life-Cycle Assessment of Apartment Buildings Based on Standard Quantities of Building Materials Using Probabilistic Analysis Technique

**DOI:** 10.3390/ma15124103

**Published:** 2022-06-09

**Authors:** Hyeonsuk Kim, Hyeongjae Jang, Sungho Tae, Hyunsik Kim, Kanghee Jo

**Affiliations:** 1 Sustainable Building Research Center, Hanyang University, 55 Hanyangdaehak-ro, Sangrok-gu, Ansan 15588, Korea; tig04160@hanyang.ac.kr; 2 Department of Architectural Engineering, Hanyang University, 55 Hanyangdaehak-ro, Sangrok-gu, Ansan 15588, Korea; visionysj@gmail.com (H.K.); rkdgml5915@gmail.com (K.J.); 3 School of Architecture and Architectural Engineering, Hanyang University, Ansan 15588, Korea; jnb55@hanyang.ac.kr

**Keywords:** probabilistic analysis techniques, building material, standard quantity, life-cycle assessment

## Abstract

Given the increasingly serious nature of environmental problems, many countries have recently declared carbon neutrality policies and expended efforts to implement them. The domestic building industry aims to reduce its environmental impact using life-cycle assessments (LCAs) of buildings according to the Green Standard for Energy and Environmental Design. However, it is difficult to perform efficient LCAs because the required quantity takeoff process is complex, and the quantity takeoff sheet may not exist during the building’s design phase. In this study, 21 building LCAs were used to simplify and improve the efficiency of the proposed method and enable building LCAs even when there was no quantity takeoff sheet. Furthermore, a standard quantity database of building materials was constructed based on the analysis of the input quantities of building materials per unit area, and the apartment buildings LCA method was proposed using this database. The input quantities of building materials were analyzed using the probabilistic analysis technique. The probability distribution was derived using Monte Carlo simulations, and the goodness-of-fit was verified. Finally, the reliability of the proposed building LCA method was verified using a case study.

## 1. Introduction

Many countries have introduced various policies to tackle increasing environmental problems and are researching ways to realize carbon neutrality [1,2]. South Korea announced the Carbon Neutrality 2050 policy in December 2020 and is working on a 3 + 1 strategy that has “reinforced the carbon-neutral institutional base” of three major policy directions: the “low carbonization of the economic structure”, the “creation of a new promising low carbon industrial ecosystem”, and a “fair transition to a carbon-neutral society” [3].

In 2017, the building and construction sector accounted for 36% of the global total energy consumption and approximately 40% of CO_2_ emissions. According to the “Paris Agreement” on a new climate regime at the 21st Conference of the Parties to the Convention on Climate Change, the South Korean Government announced a CO_2_ reduction target of 37% of the projected greenhouse gas emissions of 850.6 million tons of CO_2_eq by 2030. Additionally, based on the Amendment of the 2030 Greenhouse Gas Reduction Roadmap (July 2018), the South Korean Government raised the reduction target for the building sector to 32.7% or 64.5 million tons of CO_2_eq [4,5].

The Green Standard for Energy and Environmental Design (G-SEED) of South Korea also adopted the building life-cycle assessment (LCA) as a certification item to evaluate and reduce the environmental impact of buildings [6]. In the G-SEED, a building LCA is a bonus item worth two points. Receiving bonus points is crucial owing to the nature of G-SEED, so the frequency of building LCAs being activated is increasing. The number of G-SEED certifications has steadily increased over the past five years, from 7968 (2016), to 9733 (2017), 11,733 (2018), 13,902 (2019), and 16,225 (2020) [7]. Specifically, regarding environmental loads generated from buildings, both the energy used during the building operation phase and the environmental impact of building materials should be evaluated as important factors [8]. Currently, building LCAs define input building material quantities based on quantity takeoff sheets, which requires expert assessors. However, the information recorded in quantity takeoff sheets is limited. In addition, it is necessary to classify all the building materials into distinct categories based on the materials included in the assessment. Subsequently, the materials are grouped by collectively converting the unit weights of materials included in the assessment into tons. The final data should be organized by material segmentation according to a cumulative mass contribution cut-off of 99% for input materials. Hence, deriving the input quantities of building materials is time-consuming. Furthermore, when the assessment is performed during the architectural design phase, assessors often experience difficulties because the quantity takeoff sheet has not yet been finalized. Therefore, this study aimed to simplify the LCA process for buildings and enhance its performance even when information concerning building materials information remains unfinalized.

We constructed a database of building materials with standard quantities for apartment houses using the probabilistic analysis technique. The database was used for building material quantities input to 21 apartment houses for which G-SEED building LCAs were performed. We then proposed an LCA method for apartment buildings that uses this database. Finally, a case evaluation was performed on actual buildings.

## 2. Literature Review

LCA is an objective, environmental impact assessment method that quantifies the amount of energy and materials consumed and discharged during the life cycle of products and services, i.e., raw materials, processing, manufacturing, transportation, distribution, use, recycling, and waste management, based on the ISO 14040 series, and comprehensively evaluates their impact on the environment, and seeking ways to improve the environment based on the assessment [6,9,10,11,12,13,14]. In particular, building LCAs promote the establishment of an environmental load reduction plan for each stage of a building life cycle based on an environmental load assessment [9,10]. Hence, building LCAs, based on the evaluation of the environmental impact of the entire building process, have been adopted in various studies.

Herein, previous studies on building LCAs are reviewed and examined to obtain a rational building LCA method. Gardner et al., (2019) conducted an LCA of the Frick Environmental Center based on the Leadership in Energy and Environmental Design, a United States environmental friendliness certification system. Ben-Alon et al., (2019) determined the input material quantities of a cob earthen construction based on the International Standardization Organization (ISO) 14040 series and assessed its environmental impact by conducting an LCA [15,16]. Adalberth et al., (2001) performed an LCA for four residential buildings, while Junnila and Horvath (2003) and Kofoworola and Gheewala (2008) assessed office buildings [17,18,19]. However, these studies underwent a complex analysis process to determine building material quantities in order to reflect various building materials in the LCA and were associated with probability errors depending on the decision to include or exclude building materials based on subjective choices.

In South Korea, building LCAs have been conducted based on the G-SEED since 2016, and various studies have followed. Lim et al., (2018) collected quantity takeoff sheets to analyze quantities of inputs to buildings and converted the different units to tons for consistency. They analyzed the building material input quantities according to LCA cut-off criteria [20]. Choi et al., (2012) collected price information sheets of apartment houses in 12 complexes and converted the cost of input materials to the environmental impact unit of emission sources [21]. Thus, researchers building LCA processes based on the G-SEED in South Korea should also spend considerable time converting units of input materials. 

Globally, LCAs apply a cumulative mass contribution cut-off of 99% for materials used in buildings based on the ISO 14040 series. South Korea also performs a cut-off in building LCAs, as suggested in the G-SEED. For this, data on the quantity of building materials must be acquired, and a complex process of converting the input building materials into weights must be adopted, which is difficult. Therefore, it is necessary to simplify the LCA procedure and standardize building material quantities to replace the relevant data.

In the context of implementing LCAs, Marzouk (2019) probabilistically analyzed the input quantities of building materials based on Monte Carlo simulations. Roh et al., (2019) analyzed the input quantities of major building materials of 443 apartments using Monte Carlo simulations [22,23]. As a representative method extensively used in probabilistic analysis, Monte Carlo simulations supports effective decision-making based on a probabilistic model of variables in an uncertain situation. It simplifies the task of quantity conversion by standardizing building material input quantities using probabilistic analysis.

## 3. Materials and Methods

**Figure 1:**
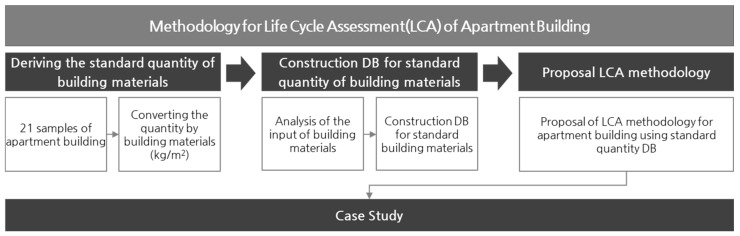
Study framework.

To propose a building LCA method using the standard building material quantities of apartment houses, we defined samples of building material quantities and constructed a standard quantity database by probabilistically analyzing quantity data. In addition, a simplified building LCA method using the constructed database was proposed, and a case study was performed to verify this method. The framework of this study is shown in Figure 1.

### 3.1. Sampling of Building Material Quantities

The Housing Act of South Korea defines apartment housing as housing structured so that each household that uses all or some of the walls, hallways, stairs, or other building facilities can reside independently within one building [24]. This study selected 21 apartment houses for which LCA was performed according to the G-SEED of South Korea among the apartment housing construction projects that satisfied these criteria. These samples consisted of apartment housings evaluated since 2017, after the implementation of the building LCA standard, and were commonly designed as reinforced concrete structures. The representative units of materials used in the quantity takeoff sheets for building materials in South Korea use different material types based on the Standard of Construction Estimate, as shown in Table 1 [25]. In this study, the building material input quantities per unit area for each sample were derived by collectively converting the units to kg/m^2^.

### 3.2. Methods of Analyzing Building Material Standard Quantities and Database Construction

The data analysis methods for standardizing building material input quantities include deterministic and probabilistic analysis techniques. The latter can determine the statistical characteristics of results, such as the minimum, maximum, expected value, and probability distribution. The assessment results can be analyzed separately using Monte Carlo simulations [23] for more accurate data analysis based on multiple sample evaluation results. Therefore, we selected the probabilistic analysis technique to establish the standard quantities of building materials.

In this study, the building materials input to construction were classified into ready-mixed concrete, reinforcing bar, section steel, glass, brick, insulator, gypsum board, cement, stone, aggregate, wood, paint, iron, and tile. The probability distribution, mean, mode, and Anderson–Darling test (A–D) statistics were determined and analyzed using the probabilistic analysis technique for the input quantities of each building material. The probability distribution of each building material was determined based on the A–D test. The goodness-of-fit was tested using the commonly used distribution fit function of the Crystal Ball in Monte Carlo simulations. The A–D test assesses the degree of agreement of the distribution shape with actual data. If the A–D statistic is smaller than 1.50, the fitness level of the probability distribution is relatively high. Subsequently, the standard quantities for each building material were selected. A database was constructed by comparing and analyzing the difference between mean and mode for each building material based on the probability distribution analysis.

### 3.3. Deriving an LCA Process for Apartment Buildings Using the Standard Quantity Database

The conventional building LCA process consists of exclusion criteria work and LCA performance processes. The former (Process #1) consists of quantity takeoff sheets for building materials, the classification of materials to be assessed, the conversion of unit weights for building materials, the grouping of building materials, building materials exclusion criteria, the subdivision of building materials, and the organization of building material data. The latter (Process #2) consists of the building information input, unit database connection, and LCA performance. This study derived the LCA process for apartment buildings using the developed standard quantity database.

## 4. Results

### 4.1. Building Material Quantity Sampling Result

The samples of 21 apartment houses were collected to analyze the standard quantities of building materials. The input quantity samples per unit area were determined by unifying the various units for each building material by converting them into weight units, as shown in Table 2.

### 4.2. Analyzed Outcomes of Standard Quantities for Building Materials and Database Construction

**Figure 2:**
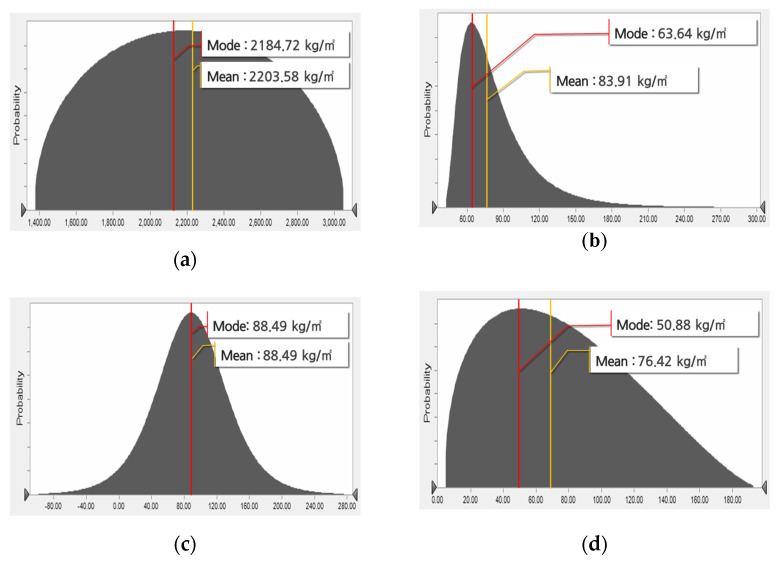
Probability distributions of representative building materials for apartment buildings. (**a**) Ready-mixed concrete. (**b**) Reinforcing bar. (**c**) Brick. (**d**) Cement.

The probability distributions of the building materials of apartment buildings are listed in Table 3. All building materials except section steel yielded A–D statistic values < 1.50, confirming the high fitness of the probability distribution. Among the 14 building materials, reinforcing bar, section steel, insulator, gypsum board, stone, aggregate, and iron, generate lognormal distributions. The glass, brick, and tile materials yielded logistic distributions, with identical means and modes. Section steel is not used in most cases because apartment houses are mostly reinforced concrete structures. Consequently, the section steel showed a relatively high A–D statistic, indicating a low fitness of probability distribution. By confirming that the A–D statistics of section steel were high, this was reflected when the standard quantity DB of section steel was finally established. The probability distribution graphs shown in Figure 2 were derived for ready-mixed concrete, reinforcing bar, brick, and cement, which are input to apartment buildings in large volumes. Figure 2 shows the difference between the minimum value and the average value derived by analyzing the building materials, and this difference can be used to improve accuracy in the building of the standard quantity DB required in this study. At this time, the X-axis of Figure 2 represents the input volume per unit area of building materials.

**Figure 3:**
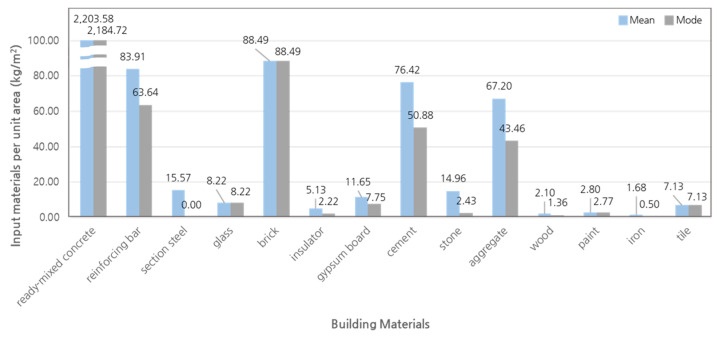
Comparison of mean and mode of building material input quantities.

A graph comparing the means and modes of all the building materials is shown in Figure 3. The difference is large in the case of most building materials, including reinforcing bar, section steel, insulator, gypsum board, cement, stone, aggregate, wood, and iron. Ready-mixed concrete, glass, brick, paint, and tile yield relatively small mean differences, and the modes are lower than 20%. The differences for reinforcing bar, gypsum board, cement, aggregate, and wood are lower than 80%. However, the differences are higher than 100% for insulator, stone, and iron. Many building materials yielded differences between the mean and mode. Therefore, the accuracy of the standard quantity database of building materials can be increased using the mode, showing a high occurrence probability.

Therefore, in this study, the mode was selected for the standard quantities of building materials using the probabilistic analysis technique. The building materials standard quantity database for apartment buildings was constructed, as shown in Table 4. The overall building process evaluation is based on the ISO 14040 series, with a cut-off of 99% of the cumulative mass contribution of building materials to be put into the building according to the standard. To this end, the cumulative mass contribution of the standard quantity of building materials constructed in this study was calculated and indicated. The representative materials for apartment buildings corresponding to the top 99% of the cumulative mass contribution based on the LCA cut-off of the ISO 14040 series are ready-mixed concrete, brick, reinforcing bar, cement, aggregate, and glass. The input quantities of iron and wood are small, at 0.50 kg/m^2^ and 1.36 kg/m^2^, respectively, while section steel is absent.

### 4.3. Contructing Proposed LCA Method for Apartment Buildings Using the Building Materials Standard Quantity Database

**Figure 4:**
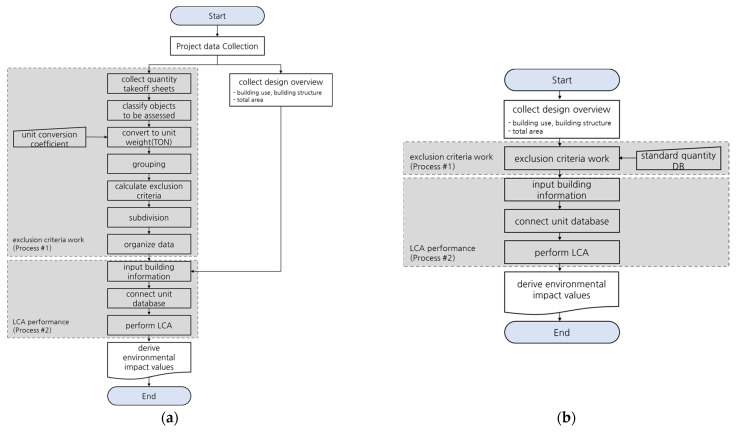
Green Standard for Energy and Environmental Design building life cycle assessment (LCA) ((**a**) conventional) and building LCA based on the standard quantity database ((**b**) this study).

The apartment building LCA process using the constructed standard quantity database is shown in Figure 4. The conversion of the unit weight of materials, the building material list analysis process in Process #1, which consumes the most time in conventional building LCAs, can be omitted if the standard quantity database developed in this study is used. Furthermore, Process #2 can be performed smoothly even if the data for input building materials is unavailable or are unclear during the building design phase by replacing the quantity takeoff sheet collection process with the standard quantity database.

## 5. Case Study

### 5.1. Overview of Case Study

To verify the reliability of this study, the outcomes of the LCA (assessment #1) based on the standard quantity database for building materials provided in this study, and the outcomes of the G-SEED building LCA (assessment #2), obtained by collecting actual quantity takeoff sheets, were determined. This case study was conducted on an apartment building located in Incheon, South Korea, and the relevant information is listed in Table 5. In assessment #1, the design overview, area, and energy requirements were collected, while in assessment #2, the design overview, area, energy requirements, and quantity takeoff sheet were collected.

### 5.2. Case Study Results

The execution of assessments #1 and #2 led to the determination of the global warming potential (GWP), abiotic depletion potential (ADP), and ozone depletion potential (ODP) for the production, construction, operation, and disuse phases of the building’s life cycle, as listed in Table 6.

For the GWP, the error rate of the results of assessments #1 and #2 is 9% and 2% in the production and construction phases, respectively. Furthermore, the results are identical in the operation phase. The error rate is high at 57% in the disuse state, but the value is small. When comparing the total environmental impact values, the error rate is lower than 4%. For the ADP, the error rate between the two assessment results in the production phase is 16%, and in the construction phase, it is 21%. Furthermore, the results in the operation phase are identical. In the disuse phase, the results using assessment #1 are smaller than those using assessment #2. However, the value is small, and the error rate is lower than 6% when comparing the total environmental impact values. For the ODP, the error rate between the two assessment results is 4% in the production and construction phases. Furthermore, the results are again identical in the operation phase. In the disuse phase, the error rate was observed to be large, at 45%, but the value is very small, and the error rate is lower than 2% when the total environmental impact values are compared.

**Figure 5:**
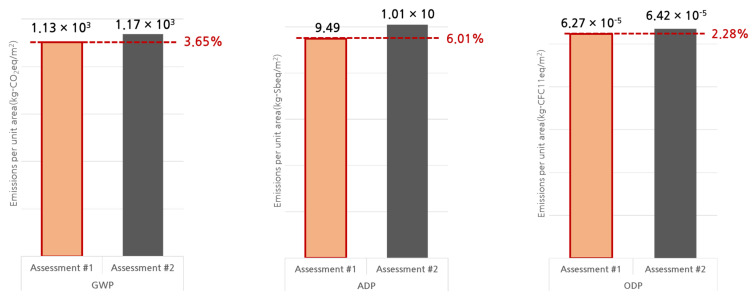
Comparison of the total life-cycle environmental impact of the apartment building.

The error rates of the GWP, ADP, and ODP obtained in assessments #1 and #2 for the total life cycle of the apartment building are lower than 4% on average, as shown in Figure 5. This means that the building LCA method proposed in this study can provide results comparable with the G-SEED building LCA that is currently executed in South Korea. This confirms that the proposed standard quantity database and building LCA method are reliable.

## 6. Discussion

The performance of building LCAs requires data concerning the quantities of input building materials, and a process must be executed for their analysis. Building LCAs using the G-SEED in South Korea are divided into the preliminary and main certification stages, performed before and at the time of building completion, respectively. In the preliminary certification stage, the quantity takeoff sheets are unavailable or are incomplete in most cases because the input quantities of building materials have not been determined. Consequently, it is difficult to receive bonus points in preliminary certification as determined by the performance of a building LCA. It is expected that this problem could be solved using the proposed LCA method.

However, the developed database is limited by the small sample size. Accordingly, there is a limit with respect to the lack of precision of the standard quantity DB. Therefore, it is necessary to derive more precise standard quantities by securing additional samples in the future. Furthermore, the featured buildings could be general buildings, business buildings, sales facilities, school facilities, accommodation facilities, or apartment buildings. The structures of Korean apartment buildings are standardized, but the other types of building have more diverse structures. Hence, there will be difficulties associated with analyzing the quantities of input materials, which requires additional research. Therefore, we plan to support the efficient performance of building LCAs by constructing an additional standard quantity database for varied buildings in the future.

## 7. Conclusions

In this study, we constructed a standard quantity database of building materials for apartment buildings to ensure a smoother and simplified building LCA process, even in the absence of data concerning the quantity of building materials, and proposed an LCA method using this database. The conclusions of this study are as follows.

1. A review of existing studies found that the building LCA process involved a complex process, namely, the analysis of input quantities of building materials, which required quantity data concerning the input of building materials. However, it is often difficult to secure accurate building material quantity data, and the analysis process is also highly complex. Therefore, the problem of data shortage could be solved and the complex assessment procedure could be simplified by standardizing the input quantities of building materials.
2. The input quantities (kg/m^2^) of each building material of each project were derived by selecting 21 apartment building projects that used the G-SEED building LCA process in South Korea as samples to standardize the building material quantities of apartment buildings.
3. The probability distribution, mean, mode, and A–D statistics of input quantities for each building material were defined using the probabilistic analysis technique. The standard quantities were then selected by comparing and analyzing the differences between the mean and mode of the building material input quantities, and a standard quantity database of apartment buildings was constructed by summarizing them.
4. A new building LCA process was proposed by replacing the takeoff sheet collection and exclusion criteria of the conventional LCA process with the standard quantity database. Thus, the building material analysis process, which is the most time-consuming process in the context of conventional assessment, could be omitted in the proposed LCA. Furthermore, the proposed LCA can be performed efficiently even when building material data are unavailable or unclear during the building design phase. A comparison of the proposed LCA and the G-SEED building LCA, which is based on actual quantity takeoff sheets, shows that the former lowered the error rate of the environmental impact per unit area by 4% on average in each building phase. This confirmed that the proposed standard quantity database and LCA method are reliable.
5. It is expected that the proposed LCA method can solve the problem associated with the use of takeoff sheets in conventional G-SEED building LCAs. However, the limitation of this study is attributed to the small number of samples selected to build the standard quantity database. In the future, it will be necessary to build a more precise standard quantity database by securing additional samples. Furthermore, we plan to support building LCAs by constructing standard quantity databases for buildings with additional uses.

## Figures and Tables

**Table 1:** Representative units of building materials.

Material	Unit	Material	Unit	Material	Unit	Material	Unit	Material	Unit
① ready-mixed concrete	m^3^	④ g lass	m^2^	⑦ gypsum board	m^2^	⑩ aggregate	m^3^	⑬ iron	m, m^2^
② reinforcing bar	m^2^, ton	⑤ brick	m^2^	⑧ cement	burlap bag	⑪ wood	m^2^	⑭ tile	m^2^
③ section steel	ton	⑥ insulator	m^2^	⑨ stone	m^2^	⑫ paint	m^2^	-	-

**Table 2:** Input quantity samples of building materials per unit area.

Project	Input Quantities of Building Materials per Unit Area (kg/m^2^)													
	① ^1^	②	③	④	⑤	⑥	⑦	⑧	⑨	⑩	⑪	⑫	⑬	⑭
#1	1864.58	61.54	0.64	7.76	106.18	1.17	9.65	71.67	64.69	29.36	1.34	0.64	0.24	8.65
#2	2146.43	92.85	18.62	7.28	66.07	2.39	8.89	79.56	4.84	47.80	0.00	4.10	3.29	4.92
#3	1637.00	67.22	0.00	6.75	72.98	4.60	18.39	62.80	4.20	56.30	3.48	1.41	1.21	8.10
#4	2500.16	115.62	0.00	10.82	91.21	14.59	6.21	91.16	14.56	82.64	0.01	3.44	1.44	7.10
#5	2413.40	81.15	0.00	8.84	114.66	21.16	4.82	109.76	6.96	62.14	10.92	2.14	1.06	11.70
#6	1917.71	58.46	0.16	0.25	100.58	2.42	14.21	105.67	11.86	125.60	1.53	3.45	1.99	9.76
#7	1857.53	84.43	0.00	12.41	258.07	1.83	5.00	12.88	0.12	40.89	2.37	1.55	0.89	6.07
#8	2248.92	75.67	0.00	16.53	111.57	3.05	5.80	21.16	10.37	64.73	0.16	1.12	0.17	9.83
#9	2622.88	95.32	7.26	4.25	62.84	2.88	11.47	64.88	0.32	190.68	3.29	4.96	1.13	6.98
#10	2287.65	66.15	11.06	6.40	109.88	3.10	8.92	38.73	2.20	127.46	2.76	0.75	2.43	5.17
#11	2976.54	161.67	0.00	5.62	68.23	3.29	18.73	93.86	70.57	41.23	1.14	2.87	4.22	1.12
#12	1489.07	47.64	0.00	20.47	22.48	3.12	3.57	26.73	11.24	71.48	0.38	2.99	0.16	6.85
#13	2644.15	89.69	0.00	10.00	118.89	3.23	10.17	115.28	12.91	32.44	0.69	5.53	1.31	6.14
#14	1619.97	54.05	0.00	10.75	25.44	11.30	25.01	42.05	4.94	66.68	1.50	3.02	0.35	5.70
#15	2323.30	145.27	0.17	7.01	50.46	4.81	10.24	23.48	14.17	62.74	2.72	2.91	3.34	6.89
#16	2281.40	77.61	0.00	6.78	79.94	4.54	10.42	74.54	8.37	29.18	3.14	3.47	0.80	7.07
#17	2848.91	100.63	0.00	6.91	174.11	6.96	8.61	77.30	4.97	73.05	2.07	1.11	0.69	8.97
#18	1626.96	54.28	0.00	10.80	25.55	3.62	25.12	42.24	5.21	66.97	1.51	3.03	0.35	5.73
#19	2272.69	67.07	0.39	8.25	85.19	4.53	14.55	166.98	20.01	49.89	2.89	4.08	1.91	5.95
#20	2742.11	87.38	0.34	8.81	75.85	3.48	12.97	138.57	26.35	65.68	2.56	2.79	3.21	5.95
#21	1953.89	65.23	0.10	0.20	119.09	2.86	9.64	145.57	8.09	16.79	1.31	2.79	3.00	15.59

^1^ Notes: ① ready-mixed concrete, ② reinforcing bar, ③ section steel, ④ glass, ⑤ brick, ⑥ insulator, ⑦ gypsum board, ⑧ cement, ⑨ stone, ⑩ aggregate, ⑪ wood, ⑫ paint, ⑬ iron, ⑭ tile.

**Table 3:** Probability distributions of various building materials.

Building Materials	Probability Distribution	Mean (kg/m^2^)	Mode (kg/m^2^)	Anderson– Darling (A–D) Value
ready-mixed concrete	beta distribution	2203.58	2184.72	0.19
reinforcing bar	lognormal distribution	75.85	63.64	0.14
section steel	lognormal distribution	15.57	0.00	2.33
glass	logistic distribution	8.22	8.22	0.43
brick	logistic distribution	88.49	88.49	0.43
insulator	lognormal distribution	5.13	2.22	0.73
gypsum board	lognormal distribution	11.65	7.75	0.27
cement	beta distribution	76.42	50.88	0.14
stone	lognormal distribution	14.96	2.43	0.39
aggregate	lognormal distribution	67.20	43.46	0.40
wood	maximum extreme distribution	2.10	1.36	0.49
paint	Weibull distribution	2.80	2.77	0.42
iron	lognormal distribution	1.68	0.50	0.38
tile	logistic distribution	7.13	7.13	0.58

**Table 4:** Apartment building material standard quantity database.

Building Materials	Standard Quantities (kg/m^2^)	Weight Contribution (%)	Cumulative Weight Contribution (%)	Representative Materials
ready-mixed concrete	2184.72	88.68	88.68	○
brick	88.49	3.59	92.27	○
reinforcing bar	63.64	2.58	94.86	○
cement	50.88	2.07	96.92	○
aggregate	43.46	1.76	98.69	○
glass	8.22	0.33	99.02	○
gypsum board	7.75	0.31	99.33	×
tile	7.13	0.29	99.62	×
paint	2.77	0.11	99.74	×
stone	2.43	0.10	99.83	×
insulator	2.22	0.09	99.92	×
wood	1.36	0.06	99.98	×
iron	0.50	0.02	100.00	×
section steel	0.00	0.00	100.00	×

**Table 5:** Case study outline.

Building Type	Apartment Building	Location	Incheon Jung-Gu, South Korea	Structure	Reinforced Concrete
Total floor area	139,442.35 m^2^	Building area	9776.05 m^2^	Exclusive use area	75,053.82 m^2^

**Table 6:** Case study results of all phases of the building’s life cycle.

Classification		Production Phase	Construction Phase	Operation Phase	Disuse Phase	Total
Global warming potential (GWP) (kg-CO_2_eq/m^2^)	assessment #1	4.84 × 10^2^	2.51× 10	6.15 × 10^2^	3.14	1.13 × 10^−3^
	assessment #2	5.30 × 10^2^	2.55 × 10	6.15 × 10^2^	2.00	1.17 × 10^3^
Abiotic depletion potential (ADP) (kg-Sbeq/m^2^)	assessment #1	1.94	1.64 × 10^−1^	7.37	1.60 × 10^−2^	9.49
	assessment #2	2.31	2.08 × 10^−1^	7.37	1.71 × 10^−1^	1.01 × 10
Ozone depletion potential (ODP) (kg-CFC11eq/m^2^)	assessment #1	2.69 × 10^−5^	6.96 × 10^−6^	2.89 × 10^−5^	1.70 × 10^−8^	6.27 × 10^−5^
	assessment #2	2.80 × 10^−5^	7.27 × 10^−6^	2.89 × 10^−5^	3.07 × 10^−8^	6.42 × 10^−5^
